# Antibody responses to COVID‐19 vaccination in people with obesity: A systematic review and meta‐analysis

**DOI:** 10.1111/irv.13078

**Published:** 2022-12-19

**Authors:** Xiaodan Ou, Jialin Jiang, Bingqian Lin, Qinyu Liu, Wei Lin, Gang Chen, Junping Wen

**Affiliations:** ^1^ Department of Endocrinology, Key Laboratory of Endocrinology, Fujian Provincial Hospital Shengli Clinical Medical College of Fujian Medical University Fuzhou China

**Keywords:** COVID‐19 vaccine, meta‐analysis, obesity

## Abstract

COVID‐19 vaccine is critical in preventing SARS‐CoV‐2 infection and transmission. However, obesity's effect on immune responses to COVID‐19 vaccines is still unknown. We performed a meta‐analysis of the literature and compared antibody responses with COVID‐19 vaccines among persons with and without obesity. We used Pubmed, Embase, Web of Science, and Cochrane Library to identify all related studies up to April 2022. The Stata.14 software was used to analyze the selected data. Eleven studies were included in the present meta‐analysis. Five of them provided absolute values of antibody titers in the obese group and non‐obese group. Overall, we found that the obese population was significantly associated with lower antibody titers (standardized mean difference [SMD] = −0.228, 95% CI [−0.437, −0.019], *P* < 0.001) after COVID‐19 vaccination. Significant heterogeneity was present in most pooled analyses but was reduced after subgroup analyses. No publication bias was observed in the present analysis. The Trim and Fill method did not change the results in the primary analysis. The present meta‐analysis suggested that obesity was significantly associated with decreased antibody responses to SARS‐CoV‐2 vaccines. Future studies should be performed to unravel the mechanism of response to the COVID‐19 vaccine in obese individuals.

## 1 INTRODUCTION

Coronavirus disease (COVID‐19) is an infectious disease caused by acute respiratory syndrome coronavirus‐2 (SARS‐CoV‐2). It is prevalent worldwide, with over 48 million confirmed cases and over 6.1 million deaths reported globally.^1^ In the present COVID‐19 pandemic, individuals with obesity have an increased risk of testing positive for SARS‐CoV‐2, with the severity of COVID‐19, and with COVID‐19 mortality.^2^, ^3^, ^4^, ^5^, ^6^ The exact mechanisms underlying the strong association between obesity and COVID‐19 were not clarified to date. At present, it has been documented that due to increased expression of proteins facilitating viral entry into cells and hyper‐glycosylation of those proteins, patients with obesity have an increased risk of becoming infected with SARS‐CoV‐2. In addition, due to an impaired pulmonary immune response, hyper‐inflammatory systemic responses, increased risk of thrombosis, and increased viral load, patients with obesity also develop severe complications upon SARS‐CoV‐2 infection with significant morbidities and mortalities.^7^ Currently, different vaccines against SARS‐CoV‐2 have been implemented worldwide to reduce COVID‐19 cases. However, obesity is associated with reduced memory immune responses.^8^ It is unknown whether the protection would be affected by this reduced immune response.

Several vaccines have been designed against the severe acute respiratory syndrome coronavirus 2 (SARS‐CoV‐2) virus, with different mechanisms of action. The main classes of coronavirus vaccines in research and development include (1) messenger ribonucleic acid (mRNA) vaccines. In December 2020, the FDA authorized the emergency use of two SARS‐CoV‐2 mRNA vaccines that utilize a 2‐dose schedule: BNT162b2/Pfizer and mRNA‐1273/Moderna.^9^, ^10^ (2) Viral vector vaccines: The Oxford‐AstraZeneca COVID‐19 vaccine, also known as AZD1222 or ChAdOx1 nCoV‐19 (ChAdOx1), was one of the earliest authorized.^11^, ^12^ (3) Inactivated vaccines and subunit vaccines: The inactivated virus cannot replicate but can still produce immunogenicity. Subunit vaccines usually contain protein or peptide antigens derived from pathogens.^13^

The efficacy of a vaccine is determined by the difference in the incidence of specific diseases among vaccinated and unvaccinated subjects for the disease. The immune responses to vaccines are evaluated by serological and/or immunological markers. The immune response could be conveniently partitioned into innate and adaptive immunity, in which the adaptive immunity is divided into 2 classes, including cell‐mediated and humoral responses.^14^ Humoral responses, only a part of immune responses, are easier to detect than other responses because of wide availability and standardization. The COVID‐19 vaccine induces detectable humoral antibodies against different antigens of severe acute SARS‐CoV‐2. One of the major immunogenic antigens in the post‐vaccine immune response is the transmembrane Spike (S), a receptor‐binding domain (RBD) that protrudes from the surface of the spherical virions and mediates virus entry into host cells.^15^ However, the antibody titers after vaccination remain significantly unpredictable, and many factors may influence it. Age,^16^ gender,^17^ and the number of doses^18^ have been shown to influence antibody titers following COVID‐19 vaccination.

Several systematic reviews of studies on COVID‐19 vaccine efficacy and effectiveness have been published.^19^, ^20^, ^21^, ^22^ Nevertheless, none focused on the efficacy of COVID‐19 vaccination in people with obesity. Therefore, the present study sought to investigate the associations between obesity and serum antibodies after COVID‐19 vaccination.

## 2 METHOD

This study was designed following the Preferred Reporting Items for Systematic Reviews and Meta‐Analyses (PRISMA) statement.^23^ The protocol of this systematic review was registered in PRISMA (CRD42022373514).

## 3 SEARCH STRATEGY

A systematic search in four online scientific databases (PubMed, EMBASE, Web of Science, and Cochrane Library) was performed in April 2022. The following search key was used in all databases without filters or restrictions: “COVID‐19 Vaccines or SARS‐CoV‐2 Vaccine” and “obesity or overweight or body mass index.” We have provided a detailed search strategy in Table S1.

## 4 SELECTION AND ELIGIBILITY CRITERIA

Four independent reviewers (XDO, JLL, BQL, and QYL) screened the titles and abstracts of identified studies for eligibility. The included studies met the following eligibility criteria: (1) Participants were previously COVID‐19 vaccinated; (2) studies included SARS‐CoV‐2 antibody levels; (3) the obesity prevalence or body mass index (BMI) data of the patients were reported. Studies were excluded for the following reasons: (1) Participants included pregnant women, cancer patients, and patients with immunodeficiency; (3) the outcomes did not include antibody titer data; (3) results were not stratified by BMI category.

## 5 DATA EXTRACTION

For eligible studies, both review authors independently extracted the data. All disagreements were resolved by the third investigator (Junping Wen). The following data were extracted from each included study: first author, publication year, country, vaccine type, number of patients in each reported BMI range, days after vaccination, history of the previous infection with COVID‐19, and outcomes. In this meta‐analysis, quality assessment of included studies was conducted using the criteria of the Newcastle‐Ottawa Scale (NOS).^24^ The NOS scores ranged from 0 to 9. A study with NOS scores ≥6 was indicated to be of high quality.

## 6 STATISTICAL ANALYSIS

A meta‐analysis was performed by Stata14.0 software (Stata Corp, College Station, TX, USA). The primary outcomes of this analysis were the titers of post‐vaccination antibodies presented as mean with standard deviation. The *I* ^2^ statistic was used to evaluate heterogeneity across the studies. When *I* ^2^ < 50%, the fixed effect model was used to combine data sets. Otherwise, the random effect model was applied. We planned to carry out the following subgroup analyses for the primary outcomes. Moreover, we conducted a subgroup meta‐analysis when heterogeneity was ultra (*I* ^2^ > 50%). The following subgroup analyses were planned for the primary outcomes: vaccine type, elapsed time since vaccination, and history of previous SARS‐CoV‐2 infection. To assess the robustness of the combined results and evaluate the effect of individual studies on this meta‐analysis, we conducted a sensitivity analysis. Subsequently, the publication bias was assessed by the Funnel Plots analysis, and Egger's regression asymmetry test was further complemented. The Trim and Fill method was used to adjust the significant publication bias.

## 7 RESULTS

**FIGURE 1:**
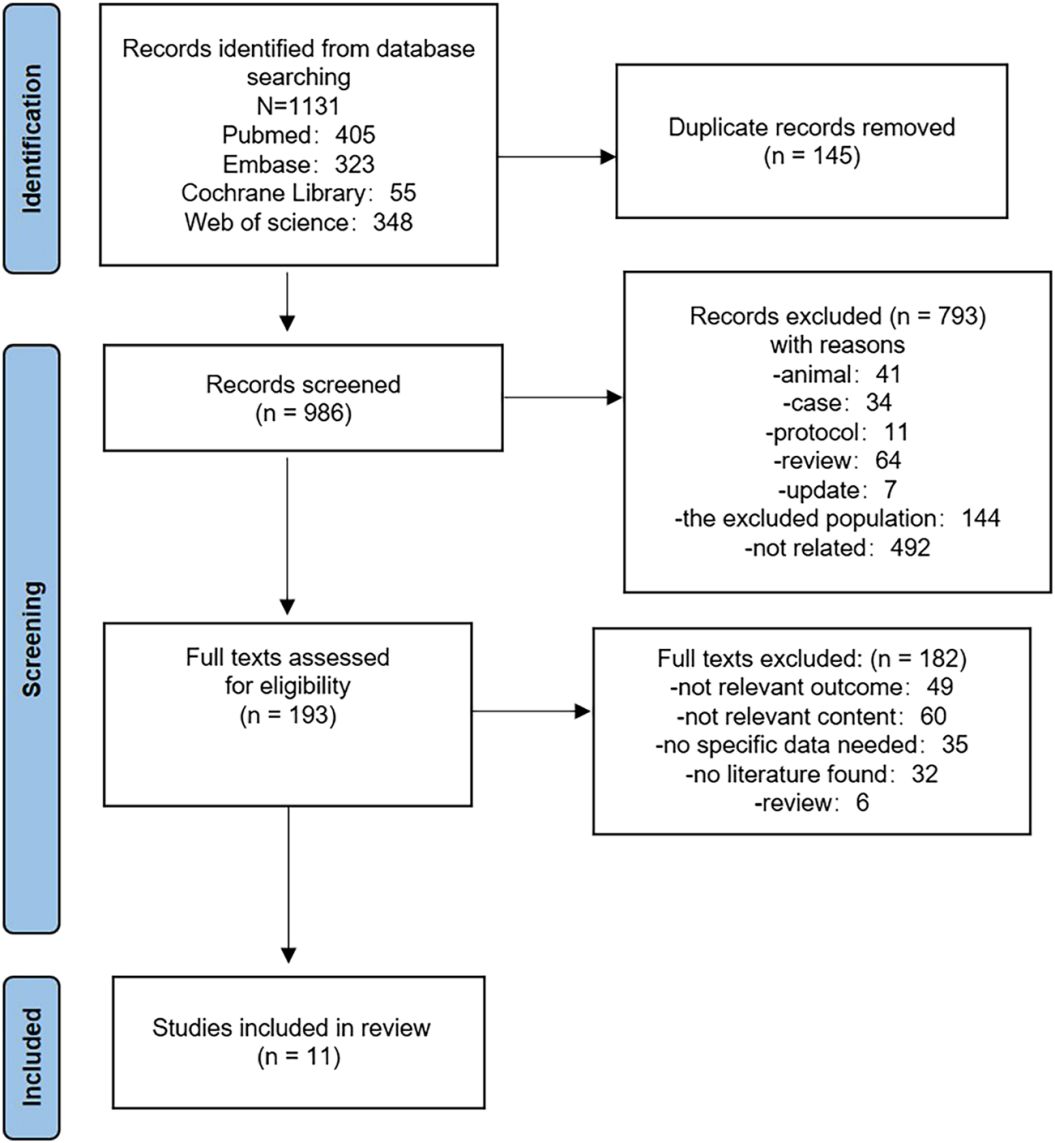
Flowchart of literature screening

We identified 1131 potentially relevant records through literature searches; 145 were duplicated articles and were excluded. After title and abstract screening, we retrieved 193 full‐text reports for further review. Finally, 11^25^, ^26^, ^27^, ^28^, ^29^, ^30^, ^31^, ^32^, ^33^, ^34^, ^35^ articles met our inclusion criteria. All included studies were of high quality (NOS > 6). The selection of the literature is summarized in Figure 1. To reduce error and explore the differences between the groups, we decided not to pool the data.

### 7.1 Characteristics of the included studies

Characteristics of participants included in the final analysis are shown in Table 1. A total of 7956 participants were included in the meta‐analysis. The age range was 18–73 years. Of these individuals, 1869 were obese. Out of 12 articles, four^25^, ^26^, ^27^, ^34^ articles reported the mRNA vaccines (BNT162b2): two^28^, ^35^ that of adenovirus‐vector vaccines (ChAdOx1 nCoV‐19) and two^29^, ^31^ that of inactivated virus vaccines (Sinovac, Sinopharm). The remaining four^31^, ^32^, ^33^ articles do not exclusively contain one type of vaccine. The range of publication years of the included studies was 2021–2022.

**TABLE 1:** Basic characteristics of the included studies

Num	Study, year	N (obesity)	Age (year)	Country	Vaccine	Assessment after administration	History of COVID‐19 infection, n	Outcome
1^27^	Alexis, 2021	1060 (492)	41.42 ± 12.95	Italy	BNT162b2	The first dose, the second dose	Yes, n = 240	① IgG neutralizing antibodies against the Trimeric complex ② The difference from the baseline titer
2^26^	Raul, 2021	248 (22)	23–69	Italy	BNT162b2	The second dose	No	① IgG antibodies against S1/S2 antigens of SARS‐CoV‐2 ② The difference from the baseline titer
3^28^	Awadhesh, 2021	552 (67)	44.8 ± 13.1	India	ChAdOx1 nCoV‐19	The first dose, the second dose	Yes, n = 60	① SARS‐CoV‐2 spike antibody titers ② Seroprevalence of antibodies
4^25^	Shohei Yamamoto, 2022	2435 (66)	/	Japan	BNT162b2	The second dose	Yes, n = 13	① Anti‐SARS‐CoV‐2 spike IgG titers
5^35^	Jer‐Hwa Chang, 2022	270 (59)	23–68	China	ChAdOx1 nCoV‐19	The first dose, the second dose	No	① anti‐RBD and anti‐spike IgG levels
6^34^	Andrea Lombardi, 2021	1218 (187)	/	Italy	BNT162b2	Second dose	Undetermined	① Seroprevalence of antibodies >7500 U/ml
7^33^	Engy Mohamed El‐Ghitany, 2022	143 (117)	43 ± 11.1	Egypt	AZD1222/BBIBP‐CorV/others	Second dose	Yes, n = 143	① Seroprevalence of anti‐S of SARS‐CoV‐2
8^32^	Rami Alqassieh, 2021	288 (249)	/	Jordan	Pfizer‐BioNTech/Sinopharm	Second dose	Yes, n = 8	① Seroprevalence of antibodies
9^31^	Xiaoguang Li, 2021	127 (44)	22–73	China	Inactivated vaccines/vector	Second dose	No	① Seroprevalence of neutralizing antibodies
10^30^	Hui Zhang, 2022	1156 (356)	18–60	China	Inactivated vaccine	Second dose	No	① Seroprevalence of neutralizing antibodies
11^29^	İlker İnanc Balkan, 2022	169 (86)	22–66	China	CoronaVac (Sinovac)	Second dose	Yes, n = 37	① Seroprevalence of antibodies

### 7.2 Relationship between obesity and antibody titers of COVID‐19 vaccination

**FIGURE 2:**
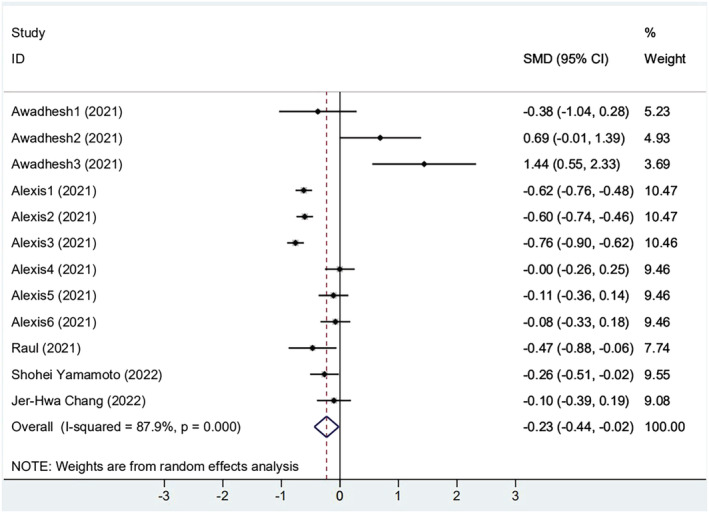
The forest plot of antibody titers

Among 11 selected studies, five^25^, ^26^, ^27^, ^28^, ^35^ studies provided absolute values of antibody titer in the obese group and non‐obese group, and two^26^, ^27^ studies offered the baseline antibody titers. The results of the meta‐analysis of the effectiveness indicator were as follows, and the random‐effects model was used for analysis: When compared with the non‐obese group, the antibody titers of the obese group were lower (SMD = −0.228, 95% CI [−0.437, −0.019], *P* < 0.001) (Figure 2). However, due to the high heterogeneity (*I* ^2^ = 87.9%), sensitivity analysis found that three subgroups of one^27^ article had the large offsets but still within the credible interval (Figure S1). We carefully reviewed the full text of this article. It was found that the population was infected with COVID‐19 previously. In addition, the following subgroups were performed in several aspects: vaccine type, elapsed time since vaccination, and history of previous SARS‐CoV‐2 infection.

**FIGURE 3:**
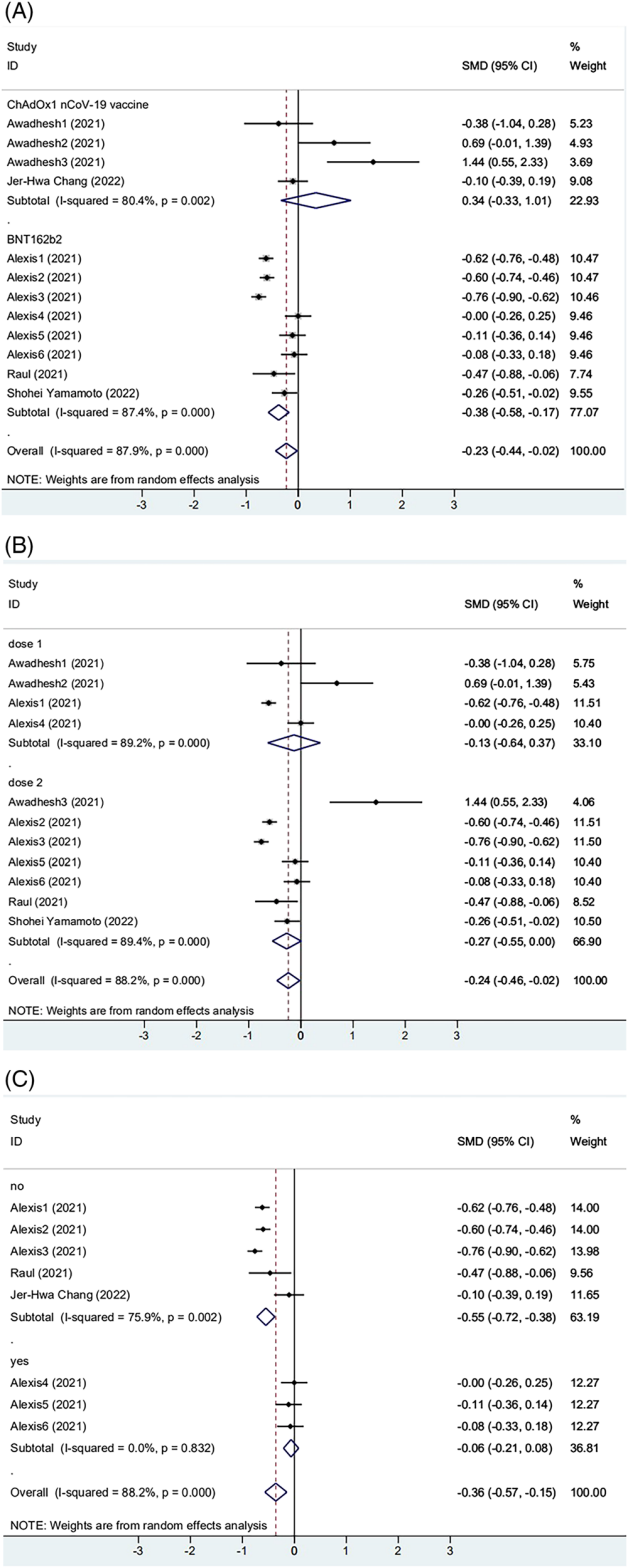
(A) The forest plot of antibody titers induced by different vaccines. (B) The forest plot of antibody titers after different doses of vaccination. (C) The forest plot of antibody titers from different populations

Among the five included articles, three^25^, ^26^, ^27^ reported BNT162b2 mRNA vaccination, and two^28^, ^35^ were vaccinated with ChAdOx1 nCoV‐19 vaccine. As the results show, both vaccinations were significantly associated with obesity (Figure 3A). However, in the participants receiving the CoronaVac vaccine, antibody titers were higher in the obese group than in the non‐obese group (SMD = 0.340, 95% CI [−0.333, 1.013], *P* = 0.002). We found that most subjects received the first dose in the group of ChAdOx1 nCoV‐19 vaccination. Next, two subgroups were designed by a different number of vaccinations: Group 1 received only one dose of vaccine; group 2 received 2 doses of vaccine. We found that two groups were significantly correlated with obesity. Antibody titers in the obese group were lower than those in the non‐obese group (Dose 1: SMD = −0.133, 95% CI [−0.636, 0.371], *P* < 0.001; Dose 2: SMD = −0.272, 95% CI [−0.548, 0.005], *P* < 0.001) (Figure 3B). It both showed lower, but the second dose was more pronounced than the first. The articles were divided into two groups according to the presence or absence of COVID‐19 infection. As shown in Figure 3C, the two groups' heterogeneity decreased. The results showed that the subgroups explained part of the heterogeneity, but notable between‐study heterogeneity still existed, so the random effect model processed the data.

**FIGURE 4:**
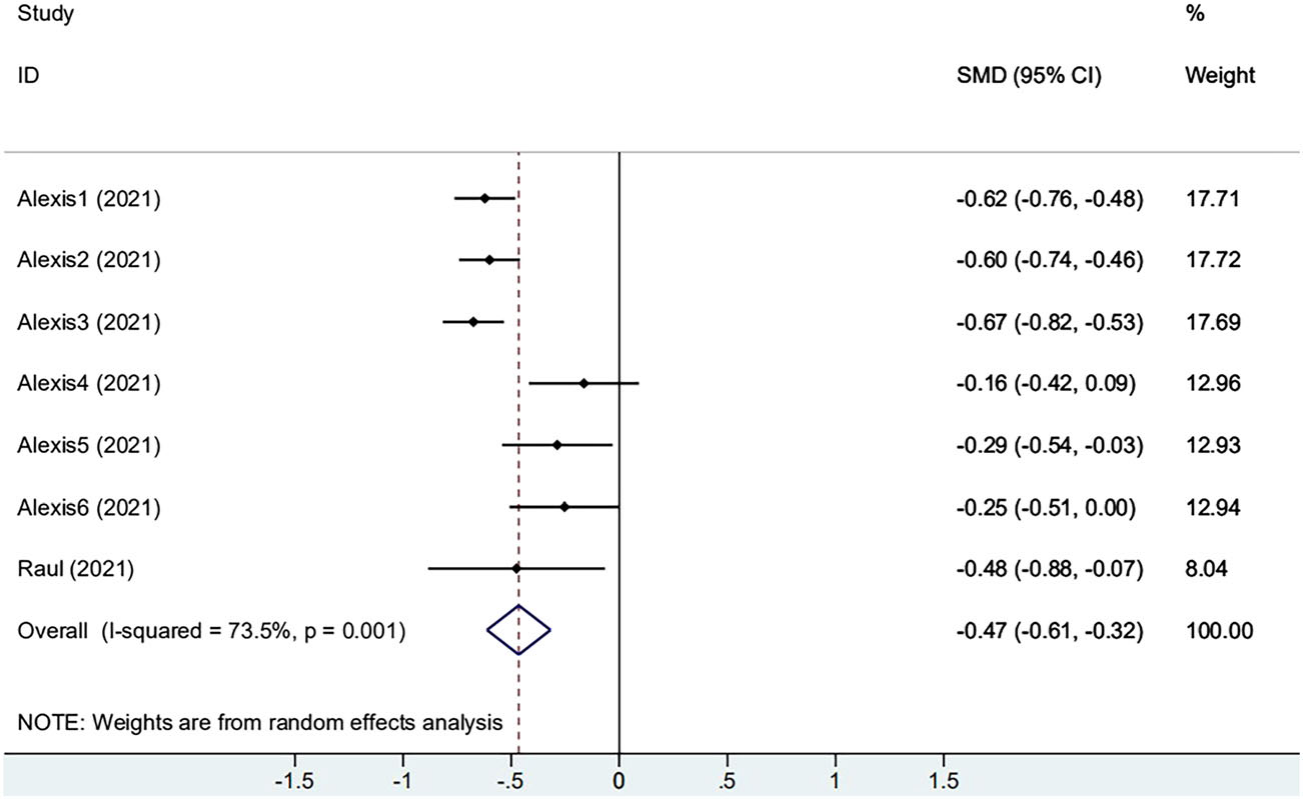
The forest plot of change from baseline of antibody titers

Two studies reported the baseline antibody titers. One of these contained six small groups. As shown in Figure 4, participants with obesity increase had a smaller increase of antibody titer compared with the non‐obese group (SMD = −0.465, 95% CI [−0.612, −0.318], *P* = 0.001), yet heterogeneity was still present (*I* ^2^ = 73.5%). Sensitivity analysis of the meta‐analysis showed that they deviated symmetrically from the center axis (Figure S2). The result indicated that patients infected with COVID‐19 already had a certain antibody level. Not only that, antibody levels in individuals with obesity were higher. The above results show that immune response to the vaccine seems to be affected by the elapsed time since vaccination and history of previous SARS‐CoV‐2 infection.

### 7.3 Relationship between obesity and seroprevalence of antibodies after COVID‐19 vaccination

**FIGURE 5:**
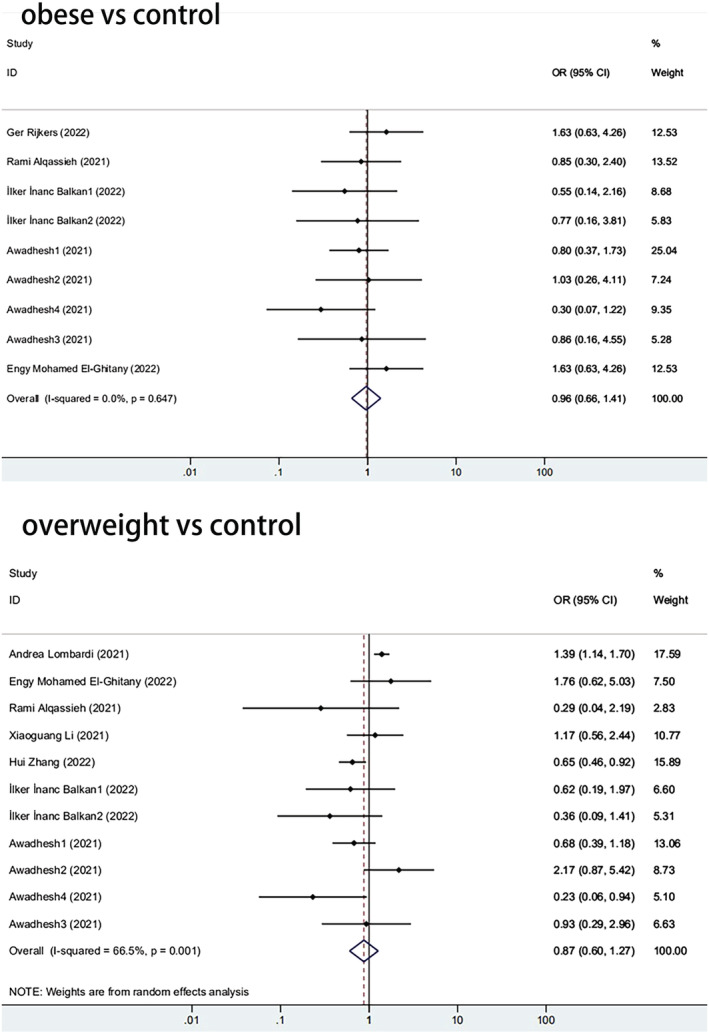
The forest plot of antibody positivity rates

Further, we analyzed how the immune response of the obese group differed from that of the control groups. Among the 11 papers, five reported differences in antibody positivity rates between obese and control groups. The antibody positivity rate of the obese was lower than that of the overall control group, with an odds ratio (OR) of 0.965 (95% CI, 0.662–1.406) (Figure 5). When BMI = 25 was used as a cut‐off value, the results showed that the immunogenicity of overweight individuals was significantly lower than that of controls (pooled OR from seven studies, 0.874; 95% CI, 0.602–1.267, *P* = 0.001) (Figure 5). The former is not statistically significant, and the latter is statistically significant, which could be attributed to the small sample size of obese groups.

### 7.4 Risk of bias of included studies

**FIGURE 6:**
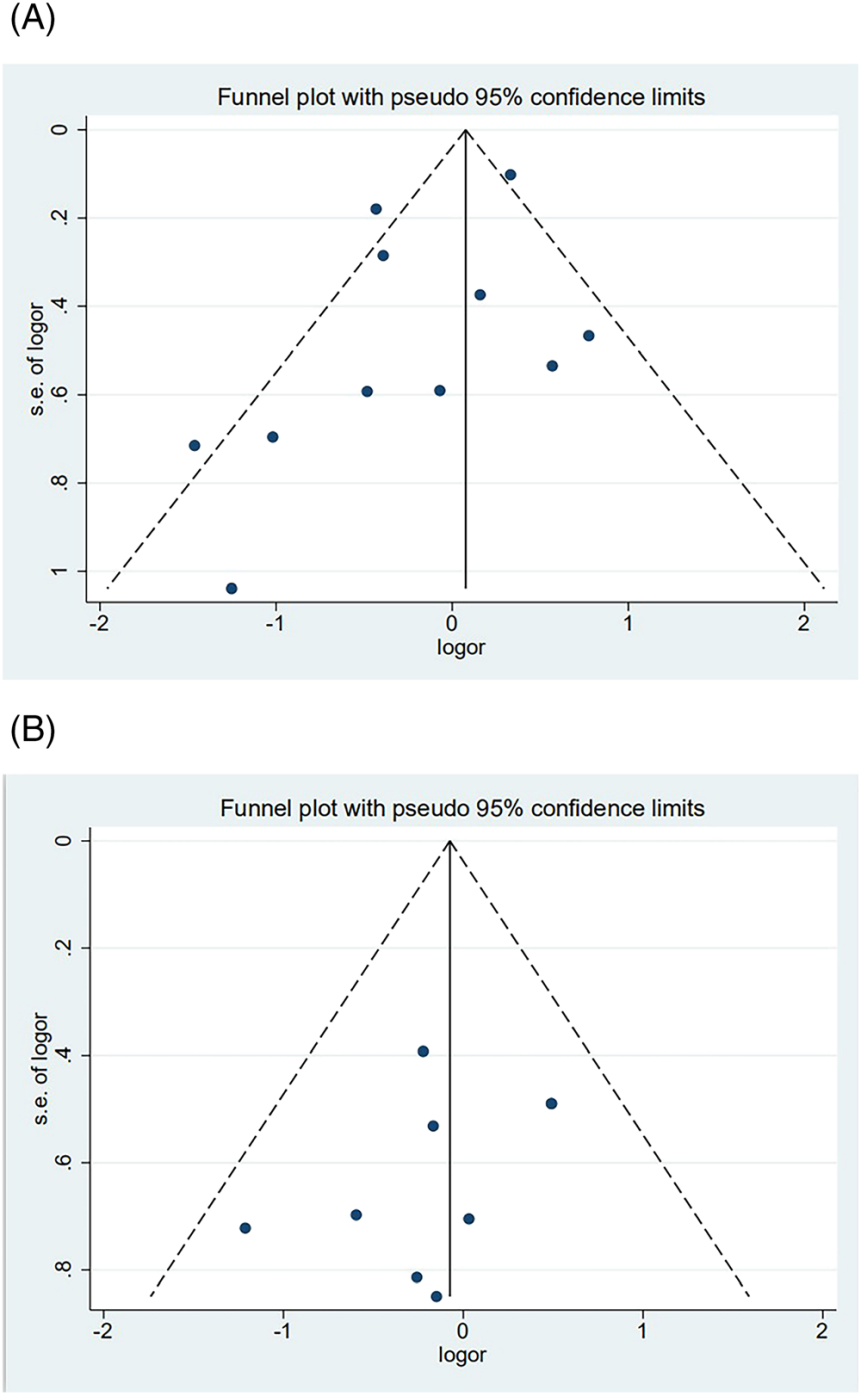
(A) The forest plot of antibody positivity rates compared with the overweight population. (B) The forest plot of antibody positivity rates compared with the obese population

**FIGURE 7:**
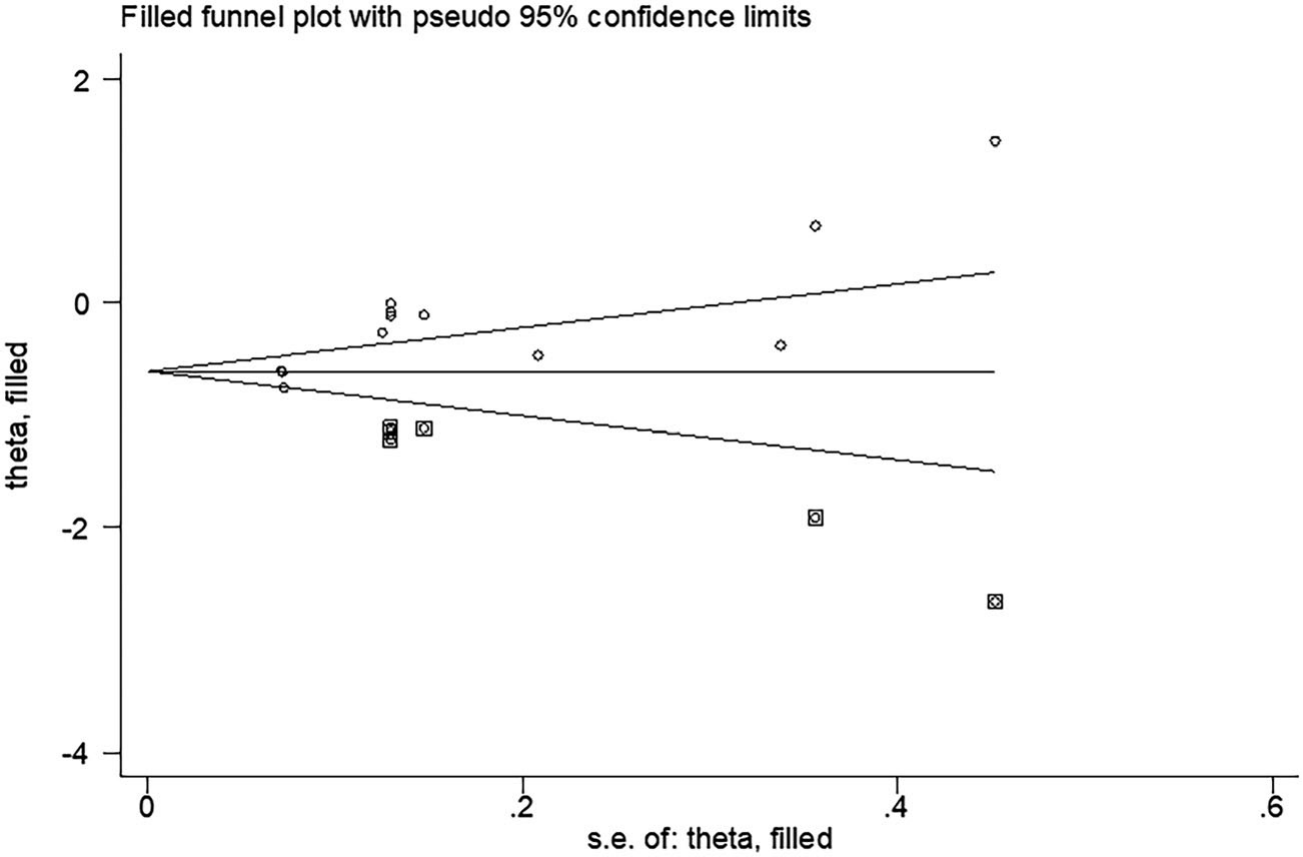
Findings of Egger's test after the Trim and Fill method

In the meta‐analysis of antibody positivity rates, the funnel plot was not strongly suggestive of publication bias (Figure 6). Findings of Egger's test (*P* = 0.290, *P* = 0.138) suggested that publication bias was not significant. Furthermore, we applied the trim‐and‐fill method to this meta‐analysis by using Metatrim. It has been presented in Figures S3 and S4, and the results showed that no trimming was performed, further suggesting no publication bias. Also, we adopted the trim‐and‐fill method to the meta‐analysis of antibody titers, and six assumed studies with favorable effects were added, and the pooled result was still robust (SMD = −0.581, 95% CI [−0.803, 0.358], *P <* 0.001) (Figure 7).

## 8 DISCUSSION

To further understand the impaired responses to COVID‐19 vaccination, more and more studies were performed to evaluate the effectiveness of the new coronavirus vaccine in different populations, including immunocompromised patients,^36^ children and adolescents,^37^ older adults,^38^ and pregnant women.^39^ This systematic review and meta‐analysis identified 11 studies evaluating the association between patients with obesity and the receipt of COVID‐19 vaccination.

In this study, our results suggested that antibody titers were low in the obese group than that in the non‐obese group (SMD = −0.228, 95% CI [−0.437, −0.019], *P* < 0.001). Moreover, compared with persons with a less than 25 of BMI, people with a BMI greater than 25 had lower antibody positivity rates after being immunized with the COVID‐19 vaccine (OR: 0.874, 95% CI: 0.602–1.267, *P* = 0.001). Additionally, visual inspection of funnel plots revealed no obvious indication of publication bias, and statistical investigation of publication bias, using the Egger intercept, was nonsignificant. In the present study of antibody titers, applying the trim‐and‐fill method by using the Trim and Fill method indicated that our conclusions were reliable because the results remained unchanged after six assumed studies. However, we observed a higher heterogeneity among 5 included studies about antibody titers. By performing subgroup analyses, we found that the heterogeneity might be caused by vaccine type, elapsed time since vaccination, and history of previous SARS‐CoV‐2 infection. Heterogeneity was lower after subgroup analysis compared with pre‐subgroup analysis.

This study shows that being overweight or obese is associated with lower antibody titers after COVID‐19 mRNA vaccination. Obesity is a metabolic problem and a chronic inflammatory disease. It increases the risk of metabolic disorders such as hyperglycemia, hyperlipidemia, hypercholesterolemia, and diabetes.^40^ Accumulating excess fat causes chronic inflammation, damages the immune system, and thus impairs antibody formation.^41^, ^42^ Accumulating excess fat causes chronic inflammation and damages the immune system, thus impairing antibody formation. The adipose tissue in obese individuals results in systemic inflammation, which increases the serum levels of diversified proinflammatory chemokines, adipokines, and cytokines. The adipose tissue in obese individuals results in systemic inflammation, which increases the serum levels of diversified proinflammatory chemokines, adipokines, and cytokines.^43^ The chronic low‐grade state of inflammation disrupts the immune response in obese persons. In obese individuals, chronic inflammation develops due to dysfunctional adipose tissue, negatively affecting T‐cell function, antibody responses, and macrophage migration.^44^ Compared with individuals with normal BMI, ACE2 receptor expression is significantly higher in adipose tissue in obese individuals.^45^, ^46^, ^47^ ACE2 is a major receptor of the spike protein of SARS‐CoV‐2, and polymorphisms of the ACE2 gene modulate the susceptibility of SARS‐CoV‐2 infection via an elevation in the expression level of ACE2.^48^ Thus, it is hypothesized that immune dysfunction increases the risk of SARS‐CoV‐2 infection and reduces vaccine response in severely obese individuals.

In addition, obesity is associated with reduced immunogenicity after hepatitis B,^49^ tetanus,^50^ and influenza^51^, ^52^, ^53^ vaccination. A recent study demonstrated that higher BMI is associated with lower Ab titers in response to the COVID‐19 vaccine in Italian healthcare workers.^54^ Thus, despite encouraging COVID‐19 vaccination results, obese patients may still be vulnerable to reinfection with SARS‐CoV‐2 long term, affecting herd immunity and SARS‐CoV‐2 elimination. In conclusion, long‐term COVID‐19 vaccine efficacy in these patients should be closely monitored to limit further effects of COVID‐19 on patients and society with obesity.

## 9 CONCLUSIONS

In summary, the present meta‐analysis suggested that obesity is significantly associated with the decreased antibody response to COVID‐19 vaccines. Patients with obesity generated significantly reduced antibody titers after COVID‐19 vaccines compared to people with normal weight. Future studies should be performed to unravel this relationship to prevent COVID‐19 infection and transmission. Our research results will lay the foundation for further meaningful research. Future studies will conduct in‐depth research on the mechanism of response to the COVID‐19 vaccine in obese individuals.

## CONFLICT OF INTEREST

Xiaodan Ou, Jialin Jiang, Bingqian Lin, Qinyu Liu, Wei Lin, Gang Chen, and Junping Wen declare that they have no conflict of interest. The authors declare no competing financial interests.

## AUTHOR CONTRIBUTIONS

**Xiaodan Ou:** Conceptualization; data curation; formal analysis; investigation; methodology; writing‐original draft. **Jialin Jiang:** Conceptualization; data curation; formal analysis. **Bingqian Lin:** Conceptualization; data curation; formal analysis. **Qinyu Liu:** Conceptualization; data curation; formal analysis. **Wei LIN:** Supervision. **Gang Chen:** Supervision. **Junping Wen:** Investigation; supervision; writing‐review and editing.

### PEER REVIEW

The peer review history for this article is available at https://publons.com/publon/10.1111/irv.13078.

## Supporting information


**Figure S1.** Sensitivity analysis of antibody titers.
**Figure S2.** Sensitivity analysis of change from baseline of antibody titers.
**Figure S3.** Egger's test of antibody positivity rates compared with overweight population.
**Figure S4.** Egger's test of antibody positivity rates compared with obese population.

**Table S1.** Details of the search history through PubMed.

## Data Availability

The data that support the findings of this study are available from the corresponding author (JPW), upon reasonable request.
