# Supplementary material for: Antibody responses to COVID‐19 vaccination in people with obesity: A systematic review and meta‐analysis
Source: Influenza Other Respir Viruses. 2022 Dec 19;17(1):e13078. doi: 10.1111/irv.13078 (PMC9835425; doi:10.1111/irv.13078)

**Supplementary figure 1. Sensitivity analysis of antibody titers.**

**
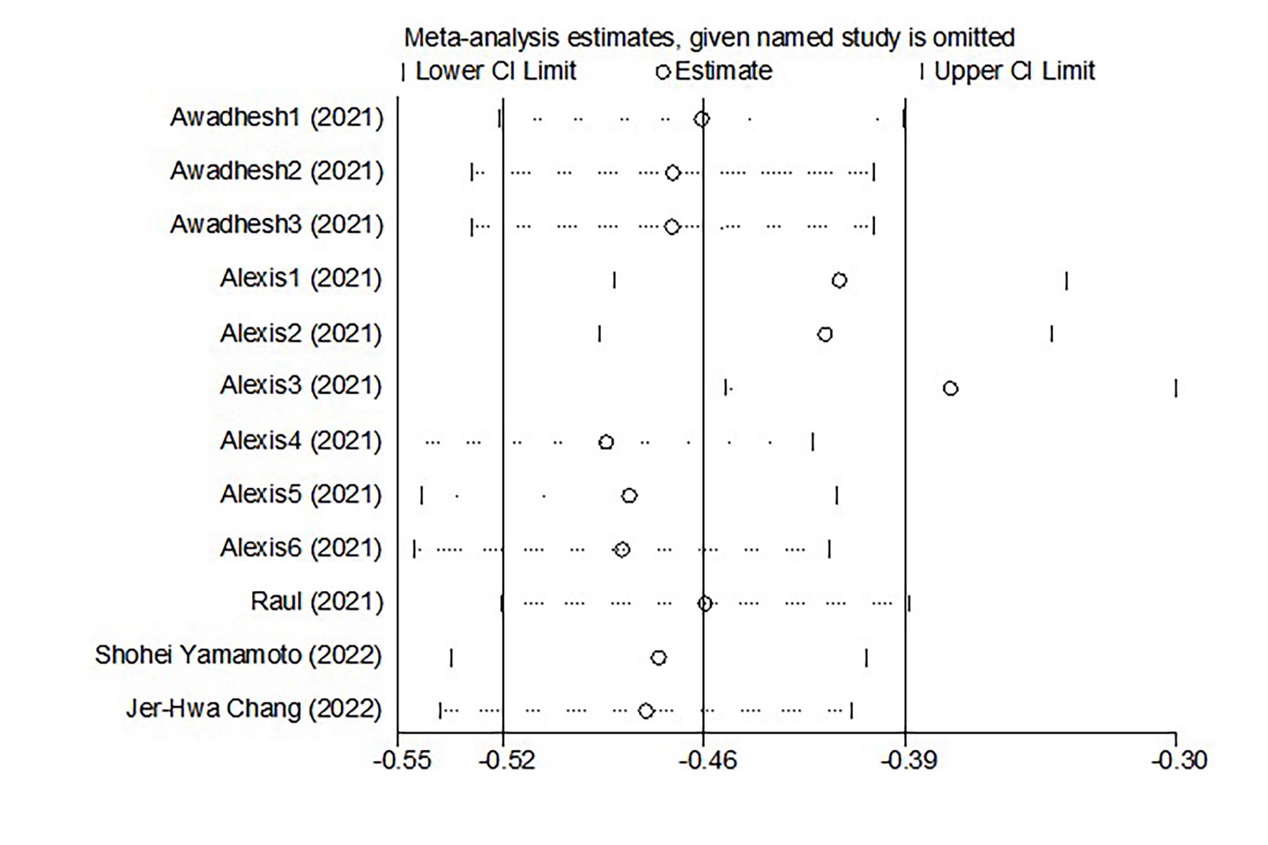
**

**Supplementary figure 2. Sensitivity analysis of change from baseline of antibody titers.**

**
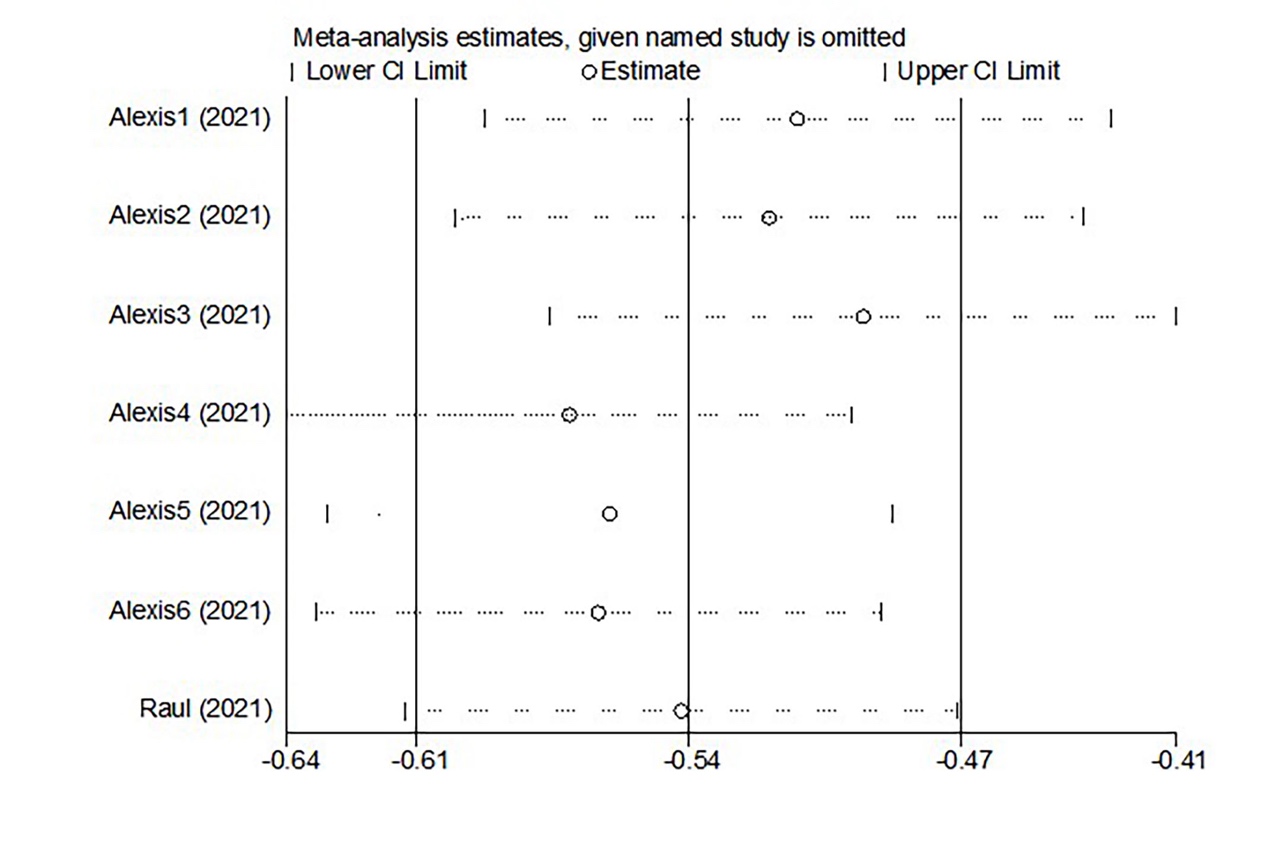
**

**Supplementary figure 3. Egger's test of antibody positivity rates compared with overweight population.**

**
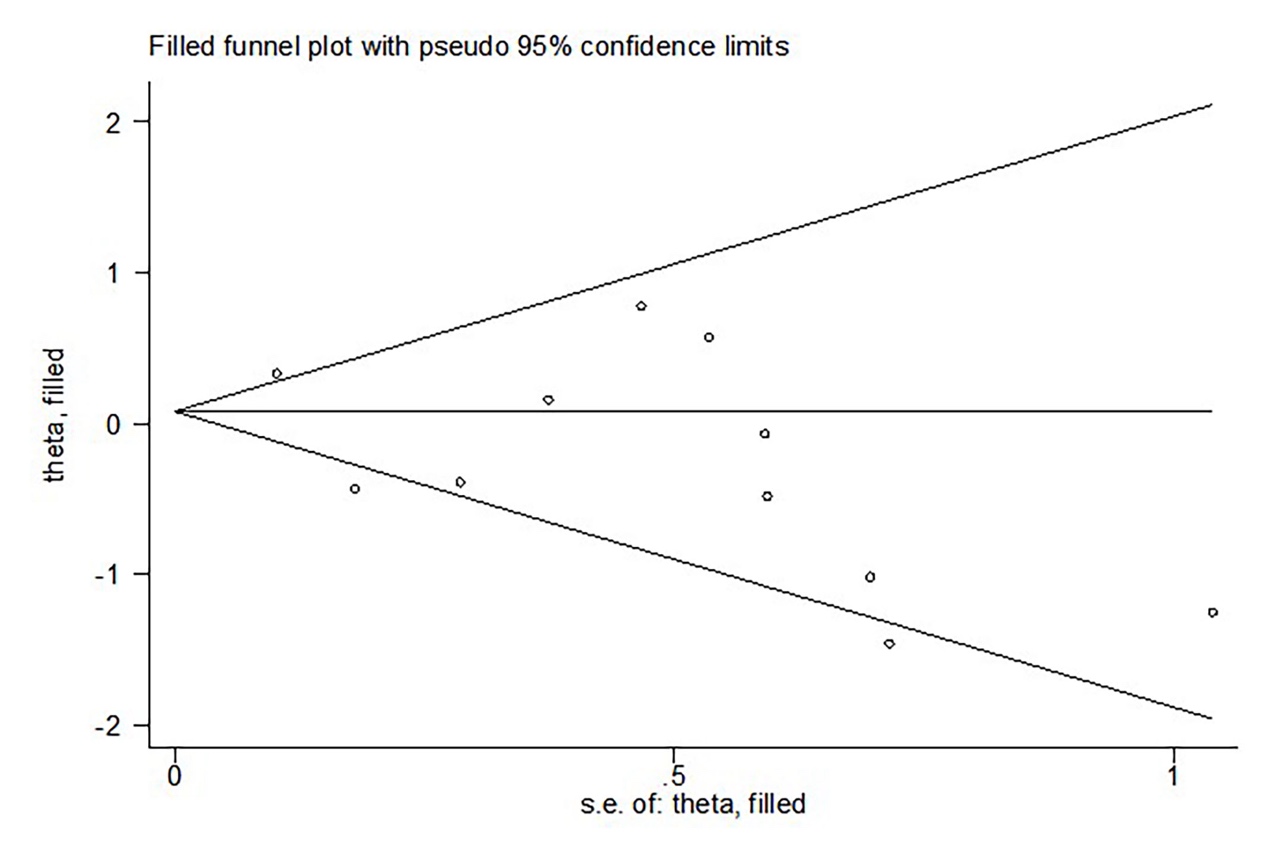
**

**Supplementary figure 4. Egger's test of antibody positivity rates compared with obese population.**


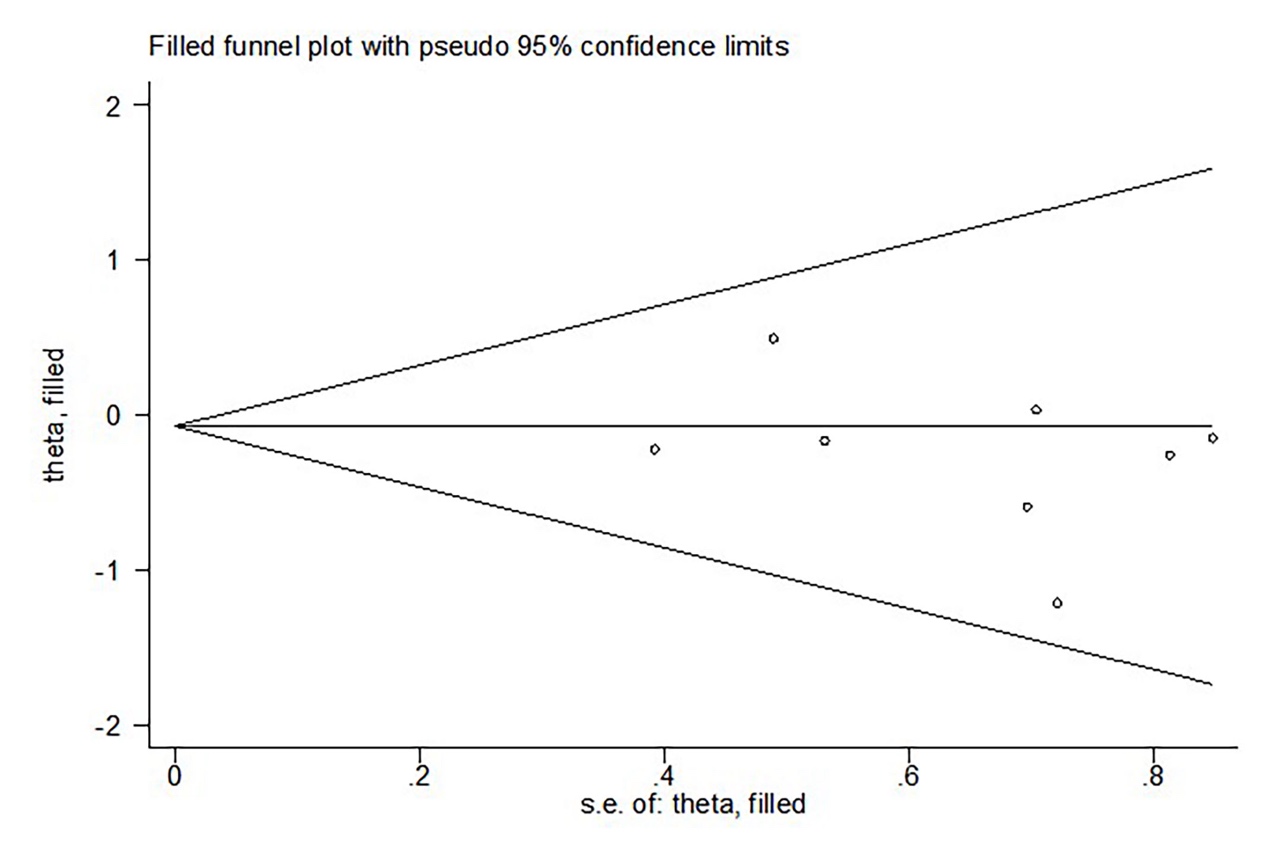

Supplement: Supplementary file 1 — Figure S1. Sensitivity analysis of antibody titers. Figure S2. Sensitivity analysis of change from baseline of antibody titers. Figure S3. Egger's test of antibody positivity rates compared with overweight population. Figure S4. Egger's test of antibody positivity rates compared with obese population. [file IRV-17-0-s001.docx]
