# Supplementary material for: Antibody responses to COVID‐19 vaccination in people with obesity: A systematic review and meta‐analysis
Source: Influenza Other Respir Viruses. 2022 Dec 19;17(1):e13078. doi: 10.1111/irv.13078 (PMC9835425; doi:10.1111/irv.13078)
Supplement: Supplementary file 2 — Table S1. Details of the search history through PubMed. [file IRV-17-0-s002.docx]

Supplementary Table 1 Details of the search history through PubMed.

| Search number | Search Details | Results |
| --- | --- | --- |
| 1 | "COVID-19 Vaccines"[MeSH Terms] | 11058 |
| 2 | "vaccines covid 19"[Title/Abstract] OR (("COVID-19"[All Fields] OR "COVID-19"[MeSH Terms] OR "covid 19 vaccines"[All Fields] OR "covid 19 vaccines"[MeSH Terms] OR "covid 19 serotherapy"[All Fields] OR "covid 19 serotherapy"[Supplementary Concept] OR "covid 19 nucleic acid testing"[All Fields] OR "covid 19 nucleic acid testing"[MeSH Terms] OR "covid 19 serological testing"[All Fields] OR "covid 19 serological testing"[MeSH Terms] OR "covid 19 testing"[All Fields] OR "covid 19 testing"[MeSH Terms] OR "SARS-CoV-2"[All Fields] OR "SARS-CoV-2"[MeSH Terms] OR "severe acute respiratory syndrome coronavirus 2"[All Fields] OR "nCoV"[All Fields] OR "2019-nCoV"[All Fields] OR (("Coronavirus"[MeSH Terms] OR "Coronavirus"[All Fields] OR "CoV"[All Fields]) AND 2019/11/01:3000/12/31[Date - Publication])) AND "virus vaccines"[Title/Abstract]) OR (("COVID-19"[All Fields] OR "COVID-19"[MeSH Terms] OR "covid 19 vaccines"[All Fields] OR "covid 19 vaccines"[MeSH Terms] OR "covid 19 serotherapy"[All Fields] OR "covid 19 serotherapy"[Supplementary Concept] OR "covid 19 nucleic acid testing"[All Fields] OR "covid 19 nucleic acid testing"[MeSH Terms] OR "covid 19 serological testing"[All Fields] OR "covid 19 serological testing"[MeSH Terms] OR "covid 19 testing"[All Fields] OR "covid 19 testing"[MeSH Terms] OR "SARS-CoV-2"[All Fields] OR "SARS-CoV-2"[MeSH Terms] OR "severe acute respiratory syndrome coronavirus 2"[All Fields] OR "nCoV"[All Fields] OR "2019-nCoV"[All Fields] OR (("Coronavirus"[MeSH Terms] OR "Coronavirus"[All Fields] OR "CoV"[All Fields]) AND 2019/11/01:3000/12/31[Date - Publication])) AND "virus vaccines"[Title/Abstract]) OR (("vaccin"[Supplementary Concept] OR "vaccin"[All Fields] OR "vaccination"[MeSH Terms] OR "vaccination"[All Fields] OR "vaccinable"[All Fields] OR "vaccinal"[All Fields] OR "vaccinate"[All Fields] OR "vaccinated"[All Fields] OR "vaccinates"[All Fields] OR "vaccinating"[All Fields] OR "vaccinations"[All Fields] OR "vaccination s"[All Fields] OR "vaccinator"[All Fields] OR "vaccinators"[All Fields] OR "vaccine s"[All Fields] OR "vaccined"[All Fields] OR "Vaccines"[MeSH Terms] OR "Vaccines"[All Fields] OR "Vaccine"[All Fields] OR "vaccins"[All Fields]) AND "covid 19 virus"[Title/Abstract]) OR "virus vaccines covid 19"[Title/Abstract] OR "covid 19 virus vaccine"[Title/Abstract] OR "covid 19 virus vaccine"[Title/Abstract] OR (("vaccin"[Supplementary Concept] OR "vaccin"[All Fields] OR "vaccination"[MeSH Terms] OR "vaccination"[All Fields] OR "vaccinable"[All Fields] OR "vaccinal"[All Fields] OR "vaccinate"[All Fields] OR "vaccinated"[All Fields] OR "vaccinates"[All Fields] OR "vaccinating"[All Fields] OR "vaccinations"[All Fields] OR "vaccination s"[All Fields] OR "vaccinator"[All Fields] OR "vaccinators"[All Fields] OR "vaccine s"[All Fields] OR "vaccined"[All Fields] OR "Vaccines"[MeSH Terms] OR "Vaccines"[All Fields] OR "Vaccine"[All Fields] OR "vaccins"[All Fields]) AND "covid 19 virus"[Title/Abstract]) OR (("virology"[MeSH Subheading] OR "virology"[All Fields] OR "viruses"[All Fields] OR "viruses"[MeSH Terms] OR "virus s"[All Fields] OR "viruse"[All Fields] OR "Virus"[All Fields]) AND "vaccine covid 19"[Title/Abstract]) OR (("COVID-19"[MeSH Terms] OR "COVID-19"[All Fields] OR "COVID19"[All Fields]) AND "virus vaccines"[Title/Abstract]) OR (("vaccin"[Supplementary Concept] OR "vaccin"[All Fields] OR "vaccination"[MeSH Terms] OR "vaccination"[All Fields] OR "vaccinable"[All Fields] OR "vaccinal"[All Fields] OR "vaccinate"[All Fields] OR "vaccinated"[All Fields] OR "vaccinates"[All Fields] | 29539 |
| 3 | "body mass index"[Title/Abstract] OR "index body mass"[Title/Abstract] OR "quetelet index"[Title/Abstract] OR "index quetelet"[Title/Abstract] OR "quetelet s index"[Title/Abstract] OR "quetelets index"[Title/Abstract] OR "Overweight"[Title/Abstract] OR ("body weights"[Title/Abstract] AND "Measures"[Title/Abstract]) OR "body size"[Title/Abstract] OR "body weight"[Title/Abstract] OR "Obesity"[Title/Abstract] OR "abdominal obesities"[Title/Abstract] OR (("obeses"[All Fields] OR "Obesity"[MeSH Terms] OR "Obesity"[All Fields] OR "obese"[All Fields] OR "Obesities"[All Fields] OR "obesity s"[All Fields]) AND "Abdominal"[Title/Abstract]) OR "central obesity"[Title/Abstract] OR "central obesities"[Title/Abstract] OR (("obeses"[All Fields] OR "Obesity"[MeSH Terms] OR "Obesity"[All Fields] OR "obese"[All Fields] OR "Obesities"[All Fields] OR "obesity s"[All Fields]) AND "Central"[Title/Abstract]) OR "obesity central"[Title/Abstract] OR "abdominal obesity"[Title/Abstract] OR "obesity visceral"[Title/Abstract] OR "visceral obesity"[Title/Abstract] OR (("obeses"[All Fields] OR "Obesity"[MeSH Terms] OR "Obesity"[All Fields] OR "obese"[All Fields] OR "Obesities"[All Fields] OR "obesity s"[All Fields]) AND "Visceral"[Title/Abstract]) OR (("Visceral"[All Fields] OR "viscerally"[All Fields]) AND "Obesities"[Title/Abstract]) OR (("Benign"[All Fields] OR "benignancies"[All Fields] OR "benignancy"[All Fields] OR "benignant"[All Fields] OR "benigne"[All Fields] OR "benignity"[All Fields] OR "benigns"[All Fields]) AND "obesity metabolically"[Title/Abstract]) OR "metabolically benign obesity"[Title/Abstract] OR "metabolically healthy obesity"[Title/Abstract] OR "healthy obesity metabolically"[Title/Abstract] OR "obesity metabolically healthy"[Title/Abstract] OR "morbid obesities"[Title/Abstract] OR (("obeses"[All Fields] OR "Obesity"[MeSH Terms] OR "Obesity"[All Fields] OR "obese"[All Fields] OR "Obesities"[All Fields] OR "obesity s"[All Fields]) AND "Morbid"[Title/Abstract]) OR "obesity severe"[Title/Abstract] OR (("obeses"[All Fields] OR "Obesity"[MeSH Terms] OR "Obesity"[All Fields] OR "obese"[All Fields] OR "Obesities"[All Fields] OR "obesity s"[All Fields]) AND "Severe"[Title/Abstract]) OR (("sever"[All Fields] OR "Severe"[All Fields] OR "severed"[All Fields] OR "severely"[All Fields] OR "severer"[All Fields] OR "severes"[All Fields] OR "severing"[All Fields] OR "severities"[All Fields] OR "severity"[All Fields] OR "severs"[All Fields]) AND "Obesities"[Title/Abstract]) OR "severe obesity"[Title/Abstract] OR "morbid obesity"[Title/Abstract] OR "obesity pediatric"[Title/Abstract] OR "obesity in childhood"[Title/Abstract] OR "childhood onset obesity"[Title/Abstract] OR "obesity childhood onset"[Title/Abstract] OR "child obesity"[Title/Abstract] OR "obesity child"[Title/Abstract] OR "childhood obesity"[Title/Abstract] OR "obesity childhood"[Title/Abstract] OR "adolescent obesity"[Title/Abstract] OR "obesity adolescent"[Title/Abstract] OR "obesity in adolescence"[Title/Abstract] OR "infantile obesity"[Title/Abstract] OR "obesity infantile"[Title/Abstract] OR "infant obesity"[Title/Abstract] OR "obesity infant"[Title/Abstract] OR "childhood overweight"[Title/Abstract] OR "overweight childhood"[Title/Abstract] OR "infant overweight"[Title/Abstract] OR "overweight infant"[Title/Abstract] OR "adolescent overweight"[Title/Abstract] OR "overweight adolescent"[Title/Abstract] | 680914 |
| 4 | ("body mass index"[Title/Abstract] OR "index body mass"[Title/Abstract] OR "quetelet index"[Title/Abstract] OR "index quetelet"[Title/Abstract] OR "quetelet s index"[Title/Abstract] OR "quetelets index"[Title/Abstract] OR "Overweight"[Title/Abstract] OR ("body weights"[Title/Abstract] AND "Measures"[Title/Abstract]) OR "body size"[Title/Abstract] OR "body weight"[Title/Abstract] OR "Obesity"[Title/Abstract] OR "abdominal obesities"[Title/Abstract] OR (("obeses"[All Fields] OR "Obesity"[MeSH Terms] OR "Obesity"[All Fields] OR "obese"[All Fields] OR "Obesities"[All Fields] OR "obesity s"[All Fields]) AND "Abdominal"[Title/Abstract]) OR "central obesity"[Title/Abstract] OR "central obesities"[Title/Abstract] OR (("obeses"[All Fields] OR "Obesity"[MeSH Terms] OR "Obesity"[All Fields] OR "obese"[All Fields] OR "Obesities"[All Fields] OR "obesity s"[All Fields]) AND "Central"[Title/Abstract]) OR "obesity central"[Title/Abstract] OR "abdominal obesity"[Title/Abstract] OR "obesity visceral"[Title/Abstract] OR "visceral obesity"[Title/Abstract] OR (("obeses"[All Fields] OR "Obesity"[MeSH Terms] OR "Obesity"[All Fields] OR "obese"[All Fields] OR "Obesities"[All Fields] OR "obesity s"[All Fields]) AND "Visceral"[Title/Abstract]) OR (("Visceral"[All Fields] OR "viscerally"[All Fields]) AND "Obesities"[Title/Abstract]) OR (("Benign"[All Fields] OR "benignancies"[All Fields] OR "benignancy"[All Fields] OR "benignant"[All Fields] OR "benigne"[All Fields] OR "benignity"[All Fields] OR "benigns"[All Fields]) AND "obesity metabolically"[Title/Abstract]) OR "metabolically benign obesity"[Title/Abstract] OR "metabolically healthy obesity"[Title/Abstract] OR "healthy obesity metabolically"[Title/Abstract] OR "obesity metabolically healthy"[Title/Abstract] OR "morbid obesities"[Title/Abstract] OR (("obeses"[All Fields] OR "Obesity"[MeSH Terms] OR "Obesity"[All Fields] OR "obese"[All Fields] OR "Obesities"[All Fields] OR "obesity s"[All Fields]) AND "Morbid"[Title/Abstract]) OR "obesity severe"[Title/Abstract] OR (("obeses"[All Fields] OR "Obesity"[MeSH Terms] OR "Obesity"[All Fields] OR "obese"[All Fields] OR "Obesities"[All Fields] OR "obesity s"[All Fields]) AND "Severe"[Title/Abstract]) OR (("sever"[All Fields] OR "Severe"[All Fields] OR "severed"[All Fields] OR "severely"[All Fields] OR "severer"[All Fields] OR "severes"[All Fields] OR "severing"[All Fields] OR "severities"[All Fields] OR "severity"[All Fields] OR "severs"[All Fields]) AND "Obesities"[Title/Abstract]) OR "severe obesity"[Title/Abstract] OR "morbid obesity"[Title/Abstract] OR "obesity pediatric"[Title/Abstract] OR "obesity in childhood"[Title/Abstract] OR "childhood onset obesity"[Title/Abstract] OR "obesity childhood onset"[Title/Abstract] OR "child obesity"[Title/Abstract] OR "obesity child"[Title/Abstract] OR "childhood obesity"[Title/Abstract] OR "obesity childhood"[Title/Abstract] OR "adolescent obesity"[Title/Abstract] OR "obesity adolescent"[Title/Abstract] OR "obesity in adolescence"[Title/Abstract] OR "infantile obesity"[Title/Abstract] OR "obesity infantile"[Title/Abstract] OR "infant obesity"[Title/Abstract] OR "obesity infant"[Title/Abstract] OR "childhood overweight"[Title/Abstract] OR "overweight childhood"[Title/Abstract] OR "infant overweight"[Title/Abstract] OR "overweight infant"[Title/Abstract] OR "adolescent overweight"[Title/Abstract] OR "overweight adolescent"[Title/Abstract]) AND ("COVID-19 Vaccines"[MeSH Terms] OR ("vaccines covid 19"[Title/Abstract] OR (("COVID-19"[All Fields] OR "COVID-19"[MeSH Terms] OR "COVID-19 Vaccines"[All Fields] OR "COVID-19 Vaccines"[MeSH Terms] OR "covid 19 serotherapy"[All Fields] OR "covid 19 serotherapy"[Supplementary Concept] OR "covid 19 nucleic acid testing"[All Fields] OR "covid 19 nucleic acid testing"[MeSH Terms] OR "covid 19 serological testing"[All Fields] OR "covid 19 serological testing"[MeSH Terms] OR "covid 19 testing"[All Fields] OR "covid 19 testing"[MeSH Terms] OR "SARS-CoV-2"[All Fields] OR "SARS-CoV-2"[MeSH Terms] OR "severe acute respiratory syndrome coronavirus 2"[All Fields] OR "nCoV"[All Fields] OR "2019-nCoV"[All Fields] OR (("Coronavirus"[MeSH Terms] OR "Coronavirus"[All Fields] OR "CoV"[All Fields]) AND 2019/11/01:3000/12/31[Date - Publication])) AND "virus vaccines"[Title/Abstract]) OR (("COVID-19"[All Fields] OR "COVID-19"[MeSH Terms] OR "COVID-19 Vaccines"[All Fields] OR "COVID-19 Vaccines"[MeSH Terms] OR "covid 19 serotherapy"[All Fields] OR "covid 19 serotherapy"[Supplementary Concept] OR "covid 19 nucleic acid testing"[All Fields] OR "covid 19 nucleic acid testing"[MeSH Terms] OR "covid 19 serological testing"[All Fields] OR "covid 19 serological testing"[MeSH Terms] OR "covid 19 testing"[All Fields] OR "covid 19 testing"[MeSH Terms] OR "SARS-CoV-2"[All Fields] OR "SARS-CoV-2"[MeSH Terms] OR "severe acute respiratory syndrome coronavirus 2"[All Fields] OR "nCoV"[All Fields] OR "2019-nCoV"[All Fields] OR (("Coronavirus"[MeSH Terms] OR "Coronavirus"[All Fields] OR "CoV"[All Fields]) AND 2019/11/01:3000/12/31[Date - Publication])) AND "virus vaccines"[Title/Abstract]) OR (("vaccin"[Supplementary Concept] OR "vaccin"[All Fields] OR "vaccination"[MeSH Terms] OR "vaccination"[All Fields] OR "vaccinable"[All Fields] OR "vaccinal"[All Fields] OR "vaccinate"[All Fields] OR "vaccinated"[All Fields] OR "vaccinates"[All Fields] OR "vaccinating"[All Fields] OR "vaccinations"[All Fields] OR "vaccination s"[All Fields] OR "vaccinator"[All Fields] OR "vaccinators"[All Fields] OR "vaccine s"[All Fields] OR "vaccined"[All Fields] OR "Vaccines"[MeSH Terms] OR "Vaccines"[All Fields] OR "Vaccine"[All Fields] OR "vaccins"[All Fields]) AND "covid 19 virus"[Title/Abstract]) OR "virus vaccines covid 19"[Title/Abstract] OR "covid 19 virus vaccine"[Title/Abstract] OR "covid 19 virus vaccine"[Title/Abstract] OR (("vaccin"[Supplementary Concept] OR "vaccin"[All Fields] OR "vaccination"[MeSH Terms] OR "vaccination"[All Fields] OR "vaccinable"[All Fields] OR "vaccinal"[All Fields] OR "vaccinate"[All Fields] OR "vaccinated"[All Fields] OR "vaccinates"[All Fields] OR "vaccinating"[All Fields] OR "vaccinations"[All Fields] OR "vaccination s"[All Fields] OR "vaccinator"[All Fields] OR "vaccinators"[All Fields] OR "vaccine s"[All Fields] OR "vaccined"[All Fields] OR "Vaccines"[MeSH Terms] OR "Vaccines"[All Fields] OR "Vaccine"[All Fields] OR "vaccins"[All Fields]) AND "covid 19 virus"[Title/Abstract]) OR (("virology"[MeSH Subheading] OR "virology"[All Fields] OR "viruses"[All Fields] OR "viruses"[MeSH Terms] OR "virus s"[All Fields] OR "viruse"[All Fields] OR "Virus"[All Fields]) AND "vaccine covid 19"[Title/Abstract]) OR (("COVID-19"[MeSH Terms] OR "COVID-19"[All Fields] OR "COVID19"[All Fields]) AND "virus vaccines"[Title/Abstract]) OR (("vaccin"[Supplementary Concept] OR "vaccin"[All Fields] OR "vaccination"[MeSH Terms] OR "vaccination"[All Fields] OR "vaccinable"[All Fields] OR "vaccinal"[All Fields] OR "vaccinate"[All Fields] OR "vaccinated"[All Fields] OR "vaccinates"[All Fields] OR "vaccinating"[All Fields] OR "vaccinations"[All Fields] OR "vaccination s"[All Fields] OR "vaccinator"[All Fields] OR "vaccinators"[All Fields] OR "vaccine s"[All Fields] OR "vaccined"[All Fields] OR "Vaccines"[MeSH Terms] OR "Vaccines"[All Fields] OR "Vaccine"[All Fields] OR "vaccins"[All Fields]) AND "covid19 virus"[Title/Abstract]) OR ((("virology"[MeSH Subheading] OR "virology"[All Fields] OR "viruses"[All Fields] OR "viruses"[MeSH Terms] OR "virus s"[All Fields] OR "viruse"[All Fields] OR "Virus"[All Fields]) AND ("vaccin"[Supplementary Concept] OR "vaccin"[All Fields] OR "vaccination"[MeSH Terms] OR "vaccination"[All Fields] OR "vaccinable"[All Fields] OR "vaccinal"[All Fields] OR "vaccinate"[All Fields] OR "vaccinated"[All Fields] OR "vaccinates"[All Fields] OR "vaccinating"[All Fields] OR "vaccinations"[All Fields] OR "vaccination s"[All Fields] OR "vaccinator"[All Fields] OR "vaccinators"[All Fields] OR "vaccine s"[All Fields] OR "vaccined"[All Fields] OR "Vaccines"[MeSH Terms] OR "Vaccines"[All Fields] OR "Vaccine"[All Fields] OR "vaccins"[All Fields])) AND "COVID19"[Title/Abstract]) OR (("COVID-19"[MeSH Terms] OR "COVID-19"[All Fields] OR "COVID19"[All Fields]) AND "virus vaccine"[Title/Abstract]) OR (("vaccin"[Supplementary Concept] OR "vaccin"[All Fields] OR "vaccination"[MeSH Terms] OR "vaccination"[All Fields] OR "vaccinable"[All Fields] OR "vaccinal"[All Fields] OR "vaccinate"[All Fields] OR "vaccinated"[All Fields] OR "vaccinates"[All Fields] OR "vaccinating"[All Fields] OR "vaccinations"[All Fields] OR "vaccination s"[All Fields] OR "vaccinator"[All Fields] OR "vaccinators"[All Fields] OR "vaccine s"[All Fields] OR "vaccined"[All Fields] OR "Vaccines"[MeSH Terms] OR "Vaccines"[All Fields] OR "Vaccine"[All Fields] OR "vaccins"[All Fields]) AND "covid19 virus"[Title/Abstract]) OR ((("virology"[MeSH Subheading] OR "virology"[All Fields] OR "viruses"[All Fields] OR "viruses"[MeSH Terms] OR "virus s"[All Fields] OR "viruse"[All Fields] OR "Virus"[All Fields]) AND ("vaccin"[Supplementary Concept] OR "vaccin"[All Fields] OR "vaccination"[MeSH Terms] OR "vaccination"[All Fields] OR "vaccinable"[All Fields] OR "vaccinal"[All Fields] OR "vaccinate"[All Fields] OR "vaccinated"[All Fields] OR "vaccinates"[All Fields] OR "vaccinating"[All Fields] OR "vaccinations"[All Fields] OR "vaccination s"[All Fields] OR "vaccinator"[All Fields] OR "vaccinators"[All Fields] OR "vaccine s"[All Fields] OR "vaccined"[All Fields] OR "Vaccines"[MeSH Terms] OR "Vaccines"[All Fields] OR "Vaccine"[All Fields] OR "vaccins"[All Fields])) AND "COVID19"[Title/Abstract]) OR "covid19 vaccines"[Title/Abstract] OR (("vaccin"[Supplementary Concept] OR "vaccin"[All Fields] OR "vaccination"[MeSH Terms] OR "vaccination"[All Fields] OR "vaccinable"[All Fields] OR "vaccinal"[All Fields] OR "vaccinate"[All Fields] OR "vaccinated"[All Fields] OR "vaccinates"[All Fields] OR "vaccinating"[All Fields] OR "vaccinations"[All Fields] OR "vaccination s"[All Fields] OR "vaccinator"[All Fields] OR "vaccinators"[All Fields] OR "vaccine s"[All Fields] OR "vaccined"[All Fields] OR "Vaccines"[MeSH Terms] OR "Vaccines"[All Fields] OR "Vaccine"[All Fields] OR "vaccins"[All Fields]) AND "COVID19"[Title/Abstract]) OR "covid19 vaccine"[Title/Abstract] OR (("vaccin"[Supplementary Concept] OR "vaccin"[All Fields] OR "vaccination"[MeSH Terms] OR "vaccination"[All Fields] OR "vaccinable"[All Fields] OR "vaccinal"[All Fields] OR "vaccinate"[All Fields] OR "vaccinated"[All Fields] OR "vaccinates"[All Fields] OR "vaccinating"[All Fields] OR "vaccinations"[All Fields] OR "vaccination s"[All Fields] OR "vaccinator"[All Fields] OR "vaccinators"[All Fields] OR "vaccine s"[All Fields] OR "vaccined"[All Fields] OR "Vaccines"[MeSH Terms] OR "Vaccines"[All Fields] OR "Vaccine"[All Fields] OR "vaccins"[All Fields]) AND "COVID19"[Title/Abstract]) OR "sars cov 2 vaccines"[Title/Abstract] OR "sars cov 2 vaccines"[Title/Abstract] OR "vaccines sars cov 2"[Title/Abstract] OR "sars cov 2 vaccine"[Title/Abstract] OR "sars cov 2 vaccine"[Title/Abstract] OR "vaccine sars cov 2"[Title/Abstract] OR (("SARS-CoV-2"[MeSH Terms] OR "SARS-CoV-2"[All Fields] OR "SARS2"[All Fields]) AND "Vaccines"[Title/Abstract]) OR (("vaccin"[Supplementary Concept] OR "vaccin"[All Fields] OR "vaccination"[MeSH Terms] OR "vaccination"[All Fields] OR "vaccinable"[All Fields] OR "vaccinal"[All Fields] OR "vaccinate"[All Fields] OR "vaccinated"[All Fields] OR "vaccinates"[All Fields] OR "vaccinating"[All Fields] OR "vaccinations"[All Fields] OR "vaccination s"[All Fields] OR "vaccinator"[All Fields] OR "vaccinators"[All Fields] OR "vaccine s"[All Fields] OR "vaccined"[All Fields] OR "Vaccines"[MeSH Terms] OR "Vaccines"[All Fields] OR "Vaccine"[All Fields] OR "vaccins"[All Fields]) AND "SARS2"[Title/Abstract]) OR (("SARS-CoV-2"[MeSH Terms] OR "SARS-CoV-2"[All Fields] OR "SARS2"[All Fields]) AND "Vaccine"[Title/Abstract]) OR (("vaccin"[Supplementary Concept] OR "vaccin"[All Fields] OR "vaccination"[MeSH Terms] OR "vaccination"[All Fields] OR "vaccinable"[All Fields] OR "vaccinal"[All Fields] OR "vaccinate"[All Fields] OR "vaccinated"[All Fields] OR "vaccinates"[All Fields] OR "vaccinating"[All Fields] OR "vaccinations"[All Fields] OR "vaccination s"[All Fields] OR "vaccinator"[All Fields] OR "vaccinators"[All Fields] OR "vaccine s"[All Fields] OR "vaccined"[All Fields] OR "Vaccines"[MeSH Terms] OR "Vaccines"[All Fields] OR "Vaccine"[All Fields] OR "vaccins"[All Fields]) AND "SARS2"[Title/Abstract]) OR "coronavirus disease 2019 vaccines"[Title/Abstract] OR "coronavirus disease 2019 vaccine"[Title/Abstract] OR (("COVID-19"[MeSH Terms] OR "COVID-19"[All Fields] OR "coronavirus disease 2019"[All Fields]) AND "virus vaccine"[Title/Abstract]) OR (("COVID-19"[MeSH Terms] OR "COVID-19"[All Fields] OR "coronavirus disease 2019"[All Fields]) AND "virus vaccines"[Title/Abstract]) OR "coronavirus disease 19 vaccines"[Title/Abstract] OR "coronavirus disease 19 vaccines"[Title/Abstract] OR (("vaccin"[Supplementary Concept] OR "vaccin"[All Fields] OR "vaccination"[MeSH Terms] OR "vaccination"[All Fields] OR "vaccinable"[All Fields] OR "vaccinal"[All Fields] OR "vaccinate"[All Fields] OR "vaccinated"[All Fields] OR "vaccinates"[All Fields] OR "vaccinating"[All Fields] OR "vaccinations"[All Fields] OR "vaccination s"[All Fields] OR "vaccinator"[All Fields] OR "vaccinators"[All Fields] OR "vaccine s"[All Fields] OR "vaccined"[All Fields] OR "Vaccines"[MeSH Terms] OR "Vaccines"[All Fields] OR "Vaccine"[All Fields] OR "vaccins"[All Fields]) AND "coronavirus disease 19"[Title/Abstract]) OR "coronavirus disease 19 vaccine"[Title/Abstract] OR "coronavirus disease 19 vaccine"[Title/Abstract] OR (("vaccin"[Supplementary Concept] OR "vaccin"[All Fields] OR "vaccination"[MeSH Terms] OR "vaccination"[All Fields] OR "vaccinable"[All Fields] OR "vaccinal"[All Fields] OR "vaccinate"[All Fields] OR "vaccinated"[All Fields] OR "vaccinates"[All Fields] OR "vaccinating"[All Fields] OR "vaccinations"[All Fields] OR "vaccination s"[All Fields] OR "vaccinator"[All Fields] OR "vaccinators"[All Fields] OR "vaccine s"[All Fields] OR "vaccined"[All Fields] OR "Vaccines"[MeSH Terms] OR "Vaccines"[All Fields] OR "Vaccine"[All Fields] OR "vaccins"[All Fields]) AND "coronavirus disease 19"[Title/Abstract]) OR "covid 19 vaccine"[Title/Abstract] OR "vaccine covid 19"[Title/Abstract] OR "2019 ncov vaccine"[Title/Abstract] OR "2019 ncov vaccine"[Title/Abstract] OR (("vaccin"[Supplementary Concept] OR "vaccin"[All Fields] OR "vaccination"[MeSH Terms] OR "vaccination"[All Fields] OR "vaccinable"[All Fields] OR "vaccinal"[All Fields] OR "vaccinate"[All Fields] OR "vaccinated"[All Fields] OR "vaccinates"[All Fields] OR "vaccinating"[All Fields] OR "vaccinations"[All Fields] OR "vaccination s"[All Fields] OR "vaccinator"[All Fields] OR "vaccinators"[All Fields] OR "vaccine s"[All Fields] OR "vaccined"[All Fields] OR "Vaccines"[MeSH Terms] OR "Vaccines"[All Fields] OR "Vaccine"[All Fields] OR "vaccins"[All Fields]) AND "2019-nCoV"[Title/Abstract]) OR ("2019"[All Fields] AND "novel coronavirus vaccines"[Title/Abstract]) OR "2019 novel coronavirus vaccine"[Title/Abstract] OR "2019 ncov vaccines"[Title/Abstract] OR "2019 ncov vaccines"[Title/Abstract] OR (("vaccin"[Supplementary Concept] OR "vaccin"[All Fields] OR "vaccination"[MeSH Terms] OR "vaccination"[All Fields] OR "vaccinable"[All Fields] OR "vaccinal"[All Fields] OR "vaccinate"[All Fields] OR "vaccinated"[All Fields] OR "vaccinates"[All Fields] OR "vaccinating"[All Fields] OR "vaccinations"[All Fields] OR "vaccination s"[All Fields] OR "vaccinator"[All Fields] OR "vaccinators"[All Fields] OR "vaccine s"[All Fields] OR "vaccined"[All Fields] OR "Vaccines"[MeSH Terms] OR "Vaccines"[All Fields] OR "Vaccine"[All Fields] OR "vaccins"[All Fields]) AND "2019-nCoV"[Title/Abstract]) OR "covid 19 vaccine"[Title/Abstract] OR "vaccine covid 19"[Title/Abstract] OR "sars coronavirus 2 vaccines"[Title/Abstract])) | 405 |
